# Quality of life and life circumstances in German myasthenia gravis patients

**DOI:** 10.1186/1477-7525-8-129

**Published:** 2010-11-11

**Authors:** Sabine Twork, Susanne Wiesmeth, Jörg Klewer, Dieter Pöhlau, Joachim Kugler

**Affiliations:** 1 Department of Health Sciences/Public Health, Faculty of Medicine "Carl Gustav Carus" at the University of Technology Dresden, Germany; 2 Department of Neurology, Kamillus-Hospital, Asbach, Germany

## Abstract

**Background:**

Myasthenia gravis (MG) is a chronic neuromuscular disease. Advances in medical therapy have continuously increased the life expectancy of MG patients, without definitively curing the disease. To analyze life circumstances and quality of life (QoL), a large German MG cohort was investigated.

**Methods and Sample:**

In cooperation with the German Myasthenia Association, 2,150 patients with confirmed MG were asked to respond to a mailed questionnaire. The standardized questions related to demographic data, impairments, therapeutic course, use of complementary therapies, illness-related costs, and quality of life (SF-36). In total, 1,518 patients participated, yielding a response rate of 70.6%. The average age was 56.7 years, and the proportion of females 58.6%.

**Results:**

Despite receiving recommended therapy, many patients still suffered from MG-related impairments. In particular, mobility and mental well-being were reduced; moreover, quality of life was markedly reduced. Stepwise linear regression analysis revealed illness stability, impairments, mental conditions, comorbid diseases, and employment to be determinants of QoL.

**Conclusion:**

Results indicate that despite prolonged life expectancy among MG patients, health-related quality of life is low. This outcome resulted mainly from impaired mobility and depression. Physical and mental well-being might be improved by additional therapy options. Additionally, health care resources could be used more efficiently in these patients.

## Background

Myasthenia gravis (MG) is a chronic, autoimmune, neuromuscular disease. The annual incidence is reported as being 0.25-4 patients per 100,000 residents, with a prevalence of 8-15 patients per 100,000 residents. The first peak of onset is around the second and third decades of life, with another one around the fifth and sixth decades. Onset of MG up to the 40^th ^year of life especially concerns women [1-6]. Myasthenic symptoms affect striated muscles. The hallmark of MG is painless, fluctuating or fatiguing weakness [7]. Patients complain early about diplopia. They often present with uni- or bilateral ptosis. Additional manifestations are bulbar symptoms such as speech and chewing disorders and dysphagia. Weakness of mimetic musculature (facies myopathica), proximal limbs, and trunk musculature can occur. In a few cases, severe muscle weakness results in respiratory failure. Typically, symptoms increase during the daytime and improve with rest [3-5]. In addition, it has been suggested that cognitive functions such as response fluency, information processing, and verbal as well as visual learning may be involved [8,9].

Nowadays, MG is graded by the MGFA Clinical Classification of Myasthenia Gravis 2002, a derivative of Osserman's and Genkins' classification system [10,11]. The pathogenetic background of the disease involves mainly antibodies (besides other types, especially acetylcholine receptor antibodies (AchR)), which impair neuromuscular transmission [1,4,6,12]. In most patients a hyperplasia of the thymus (70%-85%) and, in some cases, a thymoma (10%-15%) can be found [7,13]. According to the pathogenesis, several therapeutic strategies are applied, which range from acetylcholine esterase inhibitors such as pyridostigmine, immunosuppressors and - modulators (e.g. azathioprine, corticosteroids, methotrexate or FK506 (Tacrolimus^®^), to plasmapheresis, immunoadsorption, intravenous immunoglobulins or remove of the thymus [2-4,14-24].

Advances in medical therapy have continuously increased the life expectancy of MG patients without definitively curing the disease [3]. Within the last 10 years quality of life (QoL) aspects concerning MG were focused increasingly. MG patients often are not able to participate fully in daily life, mainly due to their muscle weakness. The persistent experience of weakness may negatively influence patients' perceived quality of life, especially among individuals for whom demands of work, family, and other responsibilities require significant physical involvement [25,26].

In 2000 a Task Force of the Myasthenia Gravis Foundation of America recommended the development of a QoL measure specific for MG. Extant investigations have relied solely on assessment of physical aspects of daily living in conceptualizing QoL ignoring important psychological factors [27]. Up to now, there are some studies (mainly clinical settings with a limited number of participants) measuring health-related QoL in MG. Tools as the widely recognized, nondisease-specific SF-36 questionnaire or the EQ-5D were applied and new, more MG-focusing questionnaires as the Myasthenia Gravis Quality of Life Scale (MG-QOL) and a shorter version, the MG-QOL15, were developed and evaluated [25-30].

The main findings of these QoL-related studies are that there is a reduction in health-related QoL, compared to normative values or control group, and this reduction is much more marked in physical domains [25,30-32].

Two retrospective studies evaluated the outcome of minimally invasive thymectomy employing health-related QoL measures (modified QoL-questionnaire of the European Organization for Research and Treatment of Cancer). They both found that patients that underwent thymectomy reported a slightly better health-related QoL compared to those that underwent a conservative approach: such a better outcome was particularly evident in younger patients and those with a lower modified Osserman score [32,33].

Three papers reported the result of a trial in which mycophenolate mofetil (MMF) was used in patients with MG [27,28,34]. The general result was that health-related QoL (SF-36) of MG patients, both on MMF and on placebo, improved in a 36-weeks period [34]. However, such an improvement was not statistically significant.

Two recent papers jointly evaluated disability and health-related QoL in MG [35,36]. They evaluated the relationships between QoL and the level of disability by relying on the SF-36 and on the World Health Organization Disability Assessment Schedule II (WHO-DAS II), whose validation has been recently published on QoL [37]. The first study reported a significant relationship between MG severity, QoL and disability profile. The second one showed significant correlations between disability and QoL, more with physical than with mental domains.

Aim of this study was to analyze precise life circumstances (e.g. impairments, therapeutic course, use of the health care system, use of complementary and alternative medicine) and resulting QoL and its determinants in a large non-clinical German cohort with confirmed MG.

## Methods

To get access to a suitable amount of data from German MG patients, a cooperation with the German Myasthenia Association, the self-help organization for MG patients in Germany, was established. All of the 2,150 patient members of this organization with confirmed MG were asked to respond anonymously to a mailed questionnaire [38,39]. The standardized questions concerned demographic data, physical and social impairments, frequency of handicapped person's pass (in Germany delivered at a certain level of impairment after proof by the "pension office" with a record concerning the degree of handicap (in %)), therapeutic course, comorbid diseases, use of the health care system, use of complementary and alternative medicine (CAM), illness-related costs, and QoL. A cover letter explained the purpose of the study to the participants, and a pre-paid envelope to return the questionnaire was included.

QoL was measured by a visual analogue scale with values from 0 to 100 and by the recognized German version of the SF-36 [40]. QoL data on the German normative sample were derived from the German National Health Survey 1998 [41]. The SF-36 is a self-administered measure of QoL that was developed to examine the impact of disease on perceived well-being. The SF-36 has been used extensively to assess QoL in patients with various diseases and has demonstrated good reliability and validity [25]. The instrument consists of 36 questions that inquire about the general health status of patients. The questions can be summarized in eight scaled categories: physical functioning (interference with physical activities), physical role (degree to which physical health necessitated change in activities during the previous four weeks), bodily pain (amount of pain experienced during the previous four weeks), general health (overall perceived health), emotional role (degree to which emotional health necessitated a change in activities during the previous four weeks), mental health (overall mood during the previous four weeks), vitality (perceived energy during the previous four weeks), and social functioning (interference with social activities) [25].

For the statistical analysis, SPSS version 16.0 was used. Applied methods were analyses of frequency (mean, median, standard deviation), t-tests for independent samples, and stepwise linear regression analysis. The research protocol of the study was carried out in accordance with the Declaration of Helsinki. All subjects received written information on the study and gave written informed consent prior to participation. An ethical approval was not necessary because there was no intervention on the patients except the survey.

## Results

### Sample

In total, 1,518 patients participated, yielding a response rate of 70.6%.

The average age of the 1,518 patients was 56.7 years (sd: 16.9 years); 38.1% of the patients were aged 65 years or older. The percentage of females was 58.6%.

Most of the patients (66.3%) were married; however, 21.5% were single, separated, or divorced, and 10.6% were widowed. Living alone was reported by 20.6% of MG patients.

Of the total, 63.1% lived in areas with fewer than 100,000 inhabitants, 20.3% in areas with 100,000-500,000 inhabitants, and 13.8% in areas with more than 500,000 inhabitants.

The overall educational level was moderate to high: 38.4% attended the "Volksschule" or "Hauptschule" (total duration of education 8-9 years), 31.6% attended the "Mittelschule" (total duration of education 10 years), and 27.1% attended the "Gymnasium" (total duration of education 12-13 years). Only 1% of the participants attended a school for special needs or did not obtain a school-completion certificate.

Ninety-two percent of the patients needed no services from nursing care insurance; however, 58.2% had a handicapped person's pass (in Germany delivered at a certain level of impairment after proof by the "pension office"), and the cohort's average degree of handicap recorded in their passes was 68%.

Nevertheless, 25.8% of the patients worked more than 15 hours per week and 3.1% fewer than 15 hours. No employment was reported by 69.2% of patients; the main reason for unemployment was age and disability related retirement.

### Disease-related data

Patients experienced their first MG symptoms at an average age of 43.6 years (sd: 19.6 years). Among women, the first symptoms occurred at an average age of 36.9 years (sd: 18.4 years), and diagnosis was made at an average age of 40.6 years (sd: 18.1 years). Among men, the average age at symptom onset was 53.4 years (sd: 16.8 years), and diagnosis was made at an average age of 55.2 years (sd: 16.1 years). The average duration of disease (from diagnosis up to the survey) was 10.2 years (sd: 9.5 years). The time from first symptoms to diagnosis (time to diagnosis) was on average 2.8 years (sd: 6.3 years). Most of the patients had a stable course of disease (81.9%).

Most of the participants had limited mobility due to increasing muscle weakness after physical strain (75.4%). Obviously, weakness of the upper limbs (71.3%) and problems in walking (69.6%) were the most impairing factors in more than two-thirds of these MG patients (table 1). About one-third of them suffered from symptoms concerning the oculofaciopharyngeal system, or from defecation problems or neck weakness. About one-fourth complained about speech disorders, facial expression disorders, and miction problems. Less-frequently reported symptoms were problems in sexuality and muscle weakness at rest.

**Table 1:** MG-related symptoms and further limitations*.

Impairments	Proportion in %
Muscle weakness after physical strain	75.4
Weakness of upper limbs	71.3
Walking problems	69.6
Dysphagia	43.9
Chewing problems	39.1
Defecation problems	38.5
Ptosis	37.8
Diplopia	37.1
Neck weakness	31.6
Speech disorders	28.9
Facial expression disorders	25.9
Miction problems	24.9
Sexual disorders	18.7
Muscle weakness at rest	16.9

* multiple answers possible

About one-third of the patients suffered from comorbid diseases such as joint and cardiac problems and depression. In about 10% of the cases, other immunological diseases were reported (table 2).

**Table 2:** Concomitant diseases in MG patients*.

Comorbid diseases	Proportion in %
Joint problems	39.4
Cardiac diseases	38.9
Depression	38.6
Hormone disorders	24.2
Osteoporosis	16.3
Metabolic disorders	12.6
Other immunological diseases	10.8
Malignancies	7.0

* multiple answers possible

### Therapy and Health care services

Nearly all patients (91.2%) had had experiences with acetylcholine esterase inhibitors; 71.4% of them had been treated with azathioprine and 55.4% with corticosteroids. A total of 58.2% of the patients had undergone a thymectomy, 14.0% used intravenous immunoglobulins, and 11.3% underwent plasmapheresis or immunoadsorption. Patients had fewer experiences with cyclosporine A (3.8%), re-thymectomy (1.4%), and radiation (4.2%).

The supervision and treatment of MG was implemented mainly by privately practicing neurologists (61.6%) or by family doctors or general practitioners (50.5%). More than one-third of the participants were treated by hospital doctors (38.8%); only 16.5% were additionally treated by privately practicing internists. Sixty-eight percent of the MG patients consulted a doctor more than six times per year, and 34.1% more than 12 times per year. A healer or non-medical practitioner was consulted by 4.2% of all patients, and 11.1% reported having further treatment by physical therapists.

Most patients (79.6%) had not received physical therapy during the previous three months; however, 9.6% received physical therapy more than six times.

In total, 87.3% of the patients received no psychotherapy. Although 13.0% of the patients were interested in receiving psychotherapy, such therapy was offered to only 11.4% of them.

Besides conventional treatment, many patients also used CAM. However, no exact definition of CAM or CAM users exists [42]. Thus, we regarded as CAM users those patients who consulted healers or non-medical practitioners, or considered themselves CAM users, or spent money on and used several alternative therapies (table 3). In total, CAM users included 40.1% of the patients (n = 609). The favorite alternative therapies were vitamins, homeopathic agents, antioxidants, and acupuncture (table 3). CAM users spent about 20 EURO (sd: 42.34 EURO, range: 5-500 EURO) monthly on homeopathic agents and about 30 EURO (sd: 51.38 EURO, range: 5-400 EURO) on other alternative therapies.

**Table 3:** CAM methods applied by 609 MG patients*.

Kind of CAM	Proportion in % (n = 609)
Vitamins	31.9
Homeopathic agents	25.6
Antioxidants	24.1
Acupuncture	23.2
Bach flowers	10.5
Special diets	9.2
Heavy metal detoxification	8.2
Bioresonance therapy	4.9
Healing stones or crystals	3.9

* multiple answers possible

### Financial burden

The overall monthly household net income was 1750.00 EURO (median, sd: 2207.25 EURO). In 54.9% of the cases, only one person contributed financially to the household's net income. Despite living under a healthcare system that covers all necessary therapies, patients paid 50 EURO (median) monthly to ease MG-related problems; 76.7% spent 25-500 EURO monthly. Such expenses were related to assistance with housekeeping and transportation as well as extra payments for prescribed medications.

In a next step, the influence of MG on patients' lives was elucidated. In 1.9% of the cases, the disease influenced the selection of school and in 8.1% the choice of job. A total of 8.5% of patients changed their job, 21.0% experienced hardships in their job, and 28.3% were forced to retire early due to MG.

### Quality of life

Through use of the SF-36, male and female MG patients were compared with each other and with a German normal population [41] regarding their health-related QoL (table 4). The SF-36 is applicable to persons aged 14 years and older [41]. Thus, data on patients aged 14 years and older (n = 1,459; females n = 886, males n = 573) were extracted from the overall cohort.

**Table 4:** Scores for each scale of the SF-36 for all 1,459 MG patients (Score range 0-100).

	females		males	
**SF-36 scale**	**MG patients** **mean (SD)**^**+**^	**normative population**^**§**^ **mean (SD)**^**+**^	**MG patients** **mean (SD)**^**+**^	**normative population**^**§**^ **mean (SD)**^**+**^

Physical functioning	56.1 (30.2)*	82.8 (22.2)	61.2 (29.3)*	88.2 (18.5)
Physical role	49.6 (42.6)	79.2 (34.8)	46.6 (43.4)*	85.5 (30.0)
Bodily pain	45.3 (15.6)	63.9 (25.9)	46.9 (15.9)	71.0 (25.3)
General health	44.6 (22.3)*	66.0 (18.7)	45.1 (22.7)*	66.8 (17.6)
Vitality	42.5 (19.6)	57.6 (18.3)	45.6 (21.9)	62.6 (17.0)
Social functioning	67.7 (28.0)	84.2 (21.2)	69.1 (28.7)*	88.6 (18.3)
Emotional role	64.0 (42.8)	86.7 (29.1)	66.0 (43.1)*	91.6 (23.8)
Mental health	62.3 (19.6)	69.8 (17.6)	64.8 (21.2)	75.2 (15.3)

* Difference from normative sample more than 1 standard deviation.

^§ ^Data by Ellert and Bellach [41].

^+ ^Standard deviation.

**Figure 1:**
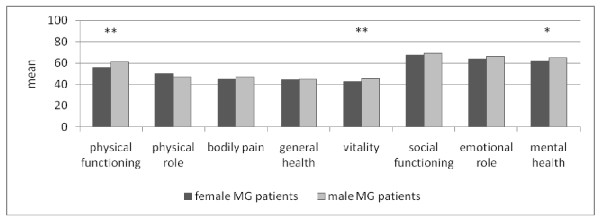
"SF-36" applied to female and male German MG patients (t-test, ** p < 0.01, * p < 0.05).

Patients evaluated their QoL, as measured on an analogue scale from 0 (extremely low) to 100 (extremely high), the average being 60.7 (sd: 23.0). The SF-36 revealed that male and female German MG patients differed significantly in physical functioning, vitality, and mental health (scores in table 4; Figure 1). The values for these three categories were lower among women. Compared with the healthy, female, German, normative population, female, German, MG patients had a decreased QoL in terms of physical functioning and general health (difference: > 1 standard deviation from normative sample). In contrast to the male, German, normative sample, male, German, MG patients additionally differed in physical and emotional role as well as social functioning (table 4).

In a next step, parameters that influence QoL were investigated by stepwise linear regression analysis. The following variables were included: age, gender, size of household (single or including several persons), size of area of residence (small towns to big cities), educational level (low to high), own expenditures due to MG (0 to > 500 EURO), employment (no or yes), monthly net income, ownership of a handicapped person's pass (no or yes), stability of MG (no or yes), duration of MG, thymectomy (no or yes), several impairing symptoms (no or yes), and comorbid diseases (no or yes) (table 5). To simplify the model, we summarized the eight categories of the SF-36 as physical health and mental health (physical health: physical functioning, physical role, bodily pain, and general health; mental health: vitality, social functioning, emotional role, and mental health; table 5).

**Table 5:** Stepwise linear regression analysis: Influences on mental and physical health.

	Physical health		Mental health	
**Variable**^**+**^	**regression coefficient**	**p**	**regression coefficient**	**P**

Age	-0.142	0.000	---	---
Employment	0.145	0.000	---	---
Own expenses due to MG	-0.136	0.000	-0.147	0.000
Duration of MG	-0.078	0.007	---	---
Stability of MG	0.155	0.000	0.200	0.000
Walking problems	-0.233	0.000	-0.118	0.000
Swallowing problems	-0.075	0.033	-0.074	0.033
Muscle weakness at rest	-0.094	0.003	---	---
Muscle weakness after physical strain	-0.123	0.000	---	---
Neck weakness	-0.094	0.004	---	---
Chewing problems	-0.078	0.029	---	---
Miction disorders	-0.100	0.001	---	---
Defecation disorders	---	---	-0.088	0.006
Facial expression disorder	---	---	-0.082	0.013
Depression	-0.160	0.000	-0.407	0.000
Other immunological diseases	-0.065	0.026		
Metabolic disorders	---	---	-0.089	0.004
Osteoporosis	---	---	-0.070	0.023
	Adjusted R^2 ^= 0.609 intercept: 76,52		Adjusted R^2 ^= 0.460 intercept: 69,78	

--- Excluded variables.

^+ ^Gender, size of household, size of area of residence, net income, handicapped person's pass, educational level, thymectomy, diplopia, ptosis, speech disorders, upper limb weakness, sexual disorders, cardiac diseases, joint problems, hormone disorders and malignancies were excluded in both calculations.

Physical health was negatively associated with walking problems, muscle weakness at rest or after physical strain, miction disorders, and depression. Parameters such as employment and a stable course of MG demonstrated a positive influence on physical health (explanation of variance = 60.9% (adjusted R^2 ^= 0.609)).

Depression, walking problems, and metabolic disorders had a negative influence on mental health, whereas stability of MG showed a positive association with mental health (explanation of variance = 46.0% (adjusted R^2 ^= 0.460)).

## Discussion

Advances in medical therapy and intensive-care technology have increased the life expectancy of MG patients [3]. Within the last 10 years QoL aspects were focused increasingly in studies on MG patients - predominantly in clinical settings with a limited number of patients (see introduction). However, up to now, scant information has emerged from population-based, non-clinical studies regarding MG patients' circumstances of life, experiences with health care and QoL. Therefore, our study focused on impairments, therapeutic course, use of the health care system, use of complementary and alternative medicine, QoL and its determinants among German MG patients.

### Study limitations

Before discussing results, several limitations of the study have to be considered.

The cooperation with the German MG self-help organization was established to address as many MG patients as possible. However, it remains unclear whether this specific, organized patient population represents the "common German MG patient".

Data used in this study were extracted from a questionnaire. On the one hand it contained well known standardizes instruments as the SF-36. On the other hand descriptions of many aspects provided by patients are based on unstandardized, pre-determined questions (e.g. presence of MG related problems).

Unfortunately, the SF-36 is a non-disease-specific QoL tool. However, by that means, a comparison to the normative German population was possible. At the time of designing the study no MG-specific QoL tools were available.

As a result of the anonymous design it was not possible to assess patients' exact clinical status of MG according to the MGFA Classification [22] or to check patients' reports with medical records. Due to the large sample size, clinical assessments of each MG patient in the whole country would have required intensive use of trained neurologists; such a procedure was impossible to implement. To obtain more information on the validity of the results, perhaps future studies should examine a portion of MG patients in order to compare assessments by experts with patients' self-reported answers. Additionally, data could be biased by the selection-bias because probably only very motivated patients took part in the survey. On the other hand, a recall-bias has to be discussed (e.g. concerning length of disease duration).

### QoL

In the following sections QoL and its determinants are discussed in the context of the international literature.

Results manifested reduced QoL in MG patients compared with a German normative cohort. Compared with normative data, Paul et al. found lower scores in seven of the eight domains of the SF-36 in MG patients (n = 27). The mean ratings concerning mental health were nearly identical to those of our study [25]. Padua et al. investigated 46 MG patients, most of them participated during periods of worsening symptoms [26,30]. The researchers found that the QoL of their patients was lowered in all scales of the SF-36, however [43].

### Determinants of QoL in MG

In our study, stepwise linear regression analysis revealed that illness stability, impairments, comorbid diseases (e.g. depression), and employment were determinants of QoL. Similar determinants of QoL were found in another German study by Winter et al. [29]: disease severity, depression, older age and increased body-mass-index (n = 37). These specific aspects are discussed more deeply in the following sections.

### Disease severity - therapy

International literature also reveals a significant relationship between MG severity groups, QoL and disability profiles [35,36]. In which way a stable or less severe disease level can be achieved? The most critical time concerning progression of MG encompasses the first 2 to 3 years after onset with an implication for early treatment. After that period, MG tends to stabilize or improve [3,44]. Spontaneous remissions even have been reported [3]. Mantegazza et al. reported the factors associated with complete, stable remission as being age at onset below 40 years, thymectomy, thymic hyperplasia, and female sex [45]. Bachmann et al. showed that patients with generalized MG who underwent thymectomy had significantly greater rates of remission and improvement compared with conservative treatment. Furthermore, they had a significantly greater survival [33].

Rates of progression of ocular MG to generalized symptoms were reported as being 49%-69% [44,46]. However, the risk of generalization can be minimized by up to 75% through treatment of patients with corticosteroids or azathioprine [46]. It has to be considered that long-term immunosuppression and thymectomy have been suspected of causing increased illness in elderly patients [44]. Thus, elderly patients require heightened attention from healthcare professionals.

Due to the problems that (1) conventional therapy for MG though does offer treatment but no complete cure and (2) immunosuppressants are linked to severe side effects, the additional therapeutic use of CAM should be considered. Our study has demonstrated the high acceptance of CAM by MG patients. However, it remains unclear whether the use of any particular CAM method results in a measurable improvement of MG-related problems. Specific literature addressing this problem is not yet available. Therefore, MG patients should be discouraged from spending a substantial amount of money on CAM.

### Impairments by MG - reduced muscle strength

Reduced muscle strength seems to be an important independent predictor for both physical and mental health.

MG patients often develop a behavioral pattern of advance planning because they have to preserve their muscle strength [47]. They have been described as sometimes avoiding social contacts due to their muscular impairments [47]. However, declines in recreational activities and social interaction are associated with decreased life satisfaction [48]. Among our patients, mobility was limited mainly by muscle weakness at rest or after physical strain, or by weakness of the upper limbs or walking difficulties. Paul et al. reported similar results in a smaller cohort with generalized MG [25].

Rostedt et al. raised the question if different types of regional muscle involvement, i.e. bulbar, ocular or generalized, in MG patients influence the mental aspects of quality of life. Bulbar and generalized involvement seems to result in an impairment of mental aspects of quality of life, whereas ocular involvement does not so [49].

Muscular weakness can be treated by drugs. The dose of acetylcholine esterase inhibitors should be well adjusted to muscular weakness. The often-practiced self-adjustment of acetylcholine esterase inhibitor's dose by patients should be supported and not strictly prohibited by physicians [47]. However, patients clearly have to be informed about potential adverse events, including possible cholinergic crisis [14]. Another approach could be encouraging MG patients to use more physical therapy. Through physical training, improvement of muscle force was reported in patients with mild MG [50]; that improvement plus optimization of gait could enhance mobility. However, little is known about the effects of physiotherapeutic methods among myasthenic patients. In one case, an exacerbation of MG during therapeutic electric stimulation was even reported [51].

However, in addition to mobility problems, decreased ability to communicate should be considered also [47]. Facial expression disorders, speech difficulties, and swallowing difficulties impair verbal and non-verbal communication [47]. Logopedic methods could ease patients' faciopharyngeal symptoms and speech difficulties, thus improving these areas. However, systematic research concerning that issue is missing.

### Comorbid diseases - mental conditions

Comorbid diseases such as depression represent another factor that influences patients' daily activities in the presented study.

All chronic diseases, including MG, may have psychiatric consequences in terms of coping and QoL. However, there are very few and partly confusing data on the prevalence and aetiology of psychiatric symptoms (e.g. depression or anxiety) in MG patients [52-54]. Depression is associated with lowered QoL in a number of chronic illnesses and might result from poor physical health along with limited activity [48].

Patients in our study showed a high rate of depression. Whether depression occurred as a result of MG or was present before the onset of MG was not determinable from our data.

We agree with Paul et al., who proposed that emotional health should remain a clinical focus [25]. Doering et al. stated that psychotherapeutic techniques may be helpful for MG patients who have psychiatric symptoms but not necessarily for MG patients in general [55]. Prospective, randomized, controlled pharmaco/psychotherapy studies are needed to better direct the management of patients and, thus, improve quality of life during the course of the illness [52].

### Employment

Despite being impaired by MG, about 30% of our patients were employed. However, working conditions do not seem to be ideal for those patients. Work-related capabilities are limited mainly by physical impairments. Occupational goals cannot be achieved, and sometimes long terms of unemployment have to be borne [47]. In our results, employment was associated with a higher QoL. However, it is not obvious whether patients who have a better state of health and thus a less impaired QoL are capable of working in the first place or whether employment itself increases QoL. The latter thesis is conceivable because employment increased QoL in patients with cancer, osteoarthritis, and spinal cord injury [48]. For that reason, adequate therapy and adjustments in the workplace are important to keep MG patients in the working environment with such positive consequences as improved health and increased net income. While at work, individuals are stimulated by physical and mental activities and social contacts. In addition, MG patients, especially young ones which face a long job as well as disease period, should be given counseling regarding future job possibilities.

### Financial burden of MG

Last but not least, despite the burden of MG on QoL also the financial burden has to be considered. Total annual costs for MG from the societal perspective in Germany were estimated by Schepelmann et al.: EUR 14,950 (95% CI 10,470-21,730) per patient. The main components of costs were the expenditures of health insurance and the loss of productivity of patients and their caregivers. Disease severity of MG and assistance in activities of the daily life were independent cost-driving factors [56].

However, there are not only costs that have to be covered by the health care system but also by the patient itself. Despite living under a healthcare system that covers neurological therapies, German MG patients incurred additional expenses due to MG (in our study about 50 EURO per month; in some cases up to 500 EURO). Such expenses involved mainly medications, housekeeping assistance, and transportation. Therefore, specific changes in the healthcare system are needed to ease burden and symptoms of MG and the related financial burden for the patient as well as the health care system.

## Conclusion

The study indicates that despite prolonged life expectancy, QoL remains reduced in MG patients. Lowered QoL results mainly from symptoms that impair mobility and psychological well-being. MG may not be a major public health problem in terms of the number of patients affected; however, as a chronic problem, it has a major financial impact on the patients themselves and the care system. MG patients spend a considerable amount of money on medications, CAM, and assistance with housekeeping, transportation, and physiotherapy--even under a healthcare system in which neurological therapy is covered by statutory public health insurance. Therefore, it follows that successful managed care of MG patients depends not only on evidence-based therapies but also on other measures that might enhance QoL. By improving mobility, psychological well-being, integration in social surroundings, the stability of disease, and the possibility of employment according to one's physical abilities, increased QoL among MG patients could be achieved. Standardized guidelines for the therapy of MG patients are recommended, in order to avoid inadequate treatment due to the rareness of the disease. Therefore, collaborative networks between general practitioners and neurologists are required to ensure proper health care for MG patients. Consequently, in the near future implementation of disease-management programs for MG patients in Germany should be considered.

## List of abbreviations

AchR: acetylcholine receptor; CAM: complementary and alternative medicine; MG: myasthenia gravis; MGFA: Myasthenia Gravis Foundation of America; MMF: mycophenolate mofetil; QoL: quality of life; sd: standard deviation.

## Competing interests

The survey was funded by the German Myasthenia Association.

There are no redundant publications and the authors declare that they have no competing interests.

## Authors' contributions

ST conceived the study, drafted its design, participated in the data collection, and drafted the manuscript. SW helped to draft the manuscript. JKl performed the statistical analysis and helped to draft the manuscript. DP and JKu participated in the design of the study and critically revised the manuscript.

All authors read and approved the final manuscript.
